# Immune Checkpoint Therapy: Tumor Draining Lymph Nodes in the Spotlights

**DOI:** 10.3390/ijms22179401

**Published:** 2021-08-30

**Authors:** Marieke F. Fransen, Thorbald van Hall, Ferry Ossendorp

**Affiliations:** 1 Department of Immunology, Leiden University Medical Center (LUMC), 2300 RC Leiden, The Netherlands; F.A.Ossendorp@lumc.nl; 2 Department of Pulmonary Diseases, Amsterdam University Medical Center, 1081 HV Amsterdam, The Netherlands; 3 Department of Medical Oncology, Leiden University Medical Center (LUMC), 2300 RC Leiden, The Netherlands; t.van_hall@lumc.nl

**Keywords:** cancer, immunotherapy, tumor-draining lymph nodes, checkpoint blockers, neoadjuvant

## Abstract

Tumor-draining lymph nodes play a paradoxical role in cancer. Surgeons often resect these sentinel lymph nodes to determine metastatic spread, thereby enabling prognosis and treatment. However, lymph nodes are vital organs for the orchestration of immune responses, due to the close encounters of dedicated immune cells. In view of the success of immunotherapy, the removal of tumor-draining lymph nodes needs to be re-evaluated and viewed in a different light. Recently, an important role for tumor-draining lymph nodes has been proposed in the immunotherapy of cancer. This new insight can change the use of immune checkpoint therapy, particularly with respect to the use in neoadjuvant settings in which lymph nodes are still operational.

## 1. Introduction

Immunotherapy with PD-1/PD-L1 blocking antibodies has reached a new phase and now moves towards application in early stage patients. For patients enrolled in therapy early after diagnosis, the administration of immune checkpoint blockers in adjuvant or neo-adjuvant settings appears very relevant. Adjuvant therapy is defined as the administration of therapeutics after the surgical resection of a tumor and/or sentinel lymph nodes (or tumor-draining lymph node TDLN). Neo-adjuvant therapy means therapeutic interception before scheduled tumor and/or TDLN resection. These two different settings may have crucial implications in immunotherapy, since immunotherapy will activate immune cells within the tumor microenvironment (TME). Therefore, the presence of the tumor will add to the potential outcome of the therapy. Moreover, the TDLN is an important organ in orchestrating immune responses. Although this is an accepted fact within the scientific tumor immunology community [1], it remains a neglected subject in recent literature. TDLN have recently been (re)discovered as key players in PD-1/PD-L1 checkpoint blockade therapy. TDLNs are resected in order to determine the stage of the disease, make informed choices on therapy and estimate the chance of distant metastases, but they have long been ignored as potentially beneficial for immunotherapy. Recent findings are bound to change that view. We and others [2,3,4,5] have shown in pre-clinical models that the TDLN plays a pivotal role in PD-1/PD-L1 blocking antibody therapy, and surgical resection prior to treatment strongly reduces therapeutic outcome [2].

In this review, we will discuss the potential of TDLN in cancer immunology and, in particular, immune checkpoint therapy, emphasizing that there is still a gap in knowledge on the immunological role of the sentinel lymph node and that it is more than a route for disseminating metastases. We argue that its role in cancer immune surveillance should be seriously reconsidered and stimulate a detailed evaluation of our systemic lymphoid draining system in the immunotherapy of cancer.

## 2. The Tumor Draining Lymph Node

The TDLN has quite extensively been investigated. However, the majority of reported studies focus on lymph angiogenesis and metastases. Clinical standards on the need for the resection of the entire lymph drainage basin of the tumor compared to single sentinel nodes have changed drastically over the last years, especially in breast cancer and melanoma, with overall survival as the main parameter [6]. However, the standards for resection can differ drastically for different tumors. The side effects of complete lymph node resection include edema, which is clearly documented. The implications of removing an organ that is essential for immune orchestration and that serves as a reservoir for activated immune cells, however, are not considered.

Up until the breakthrough of the clinical success of the immune checkpoint blockade, TDLN were studied regularly for anti-tumor immune response factors. Since the mechanistic effects of the immune checkpoint blockade are generally assumed to rely on the reactivation of anti-tumor T cells, all focus has shifted to the TME, while the TDLN received less attention. The relevant insights and analyses made on the differences between TDLN and non-draining LN (NDLN) from before checkpoint therapy are expertly reviewed by Cochran et al. [7]. The specific changes between TDLN compared to other LN are described here and include the cross-presentation of tumor-antigen, the abundance of tumor-specific T cells and a strong level of immune suppression, as defined by higher numbers of regulatory T cells, levels of IDO, TGF-β and other suppressive factors. A few recent studies performed on animal- and patient-derived TDLN revealed an important role in the onset of the anti-tumor immune response, with tumor-antigen cross-presentation taking place in the TDLN and higher levels of tumor-specific T cells found in TDLN versus NDLN [8]. Furthermore, before the clinical success of immunotherapy, the sentinel lymph node was studied as a potential source of tumor-specific T cells to be employed for adoptive cell transfer [9]. Notwithstanding the success of the adoptive cell therapy of Tumor infiltrating lymphocytes (TILs), TDLN might still constitute an important source of tumor-specific T cells [10]. Whether this approach is valuable for pursuit in the clinic is beyond the scope of this review, but we would like to stress that this fact proves that there is anti-tumor potential within TDLN, which can be used for the benefit of patients.

Besides serving as a potential source for adoptive transfer, the targets for checkpoint blockade therapy, the most potent and successful immunotherapy at the moment, can demonstrably be detected within TDLN. Both in pre-clinical mouse models of cancer and patient samples, PD-L1 and PD-1 have been found to be expressed in TDLN more extensively than in NDLN [2,3,11]. And in a recent study, the NK cells within TDLN expressed different markers compared to NDLN, including NKG2A, which has been shown to have checkpoint qualities, and can lead to activation upon blockade. These activated NK cells displayed anti-tumor activity more vigorously than NK cells from NDLN in both mouse models and breast cancer patient biopsies [12].

**Figure 1:**
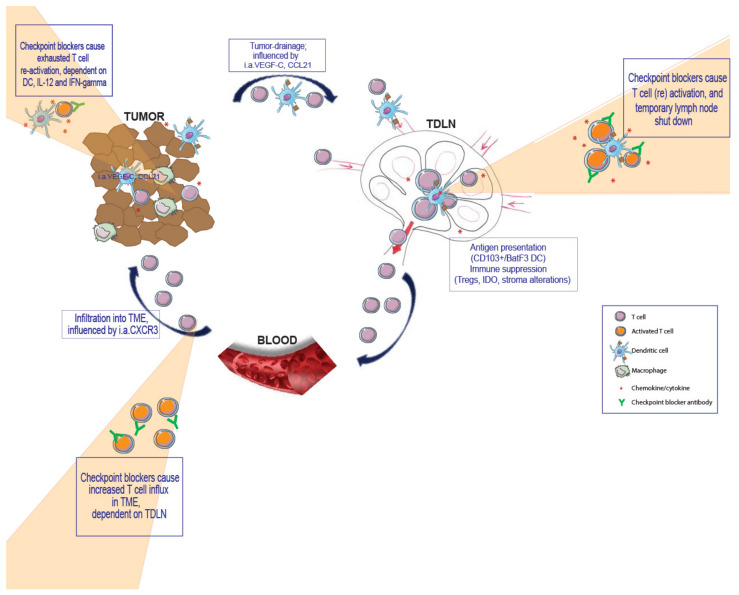
Schematic representation of the proposed working mechanism of checkpoint blocker antibodies for the therapy of cancer. Illustration made using images from Servier (https://smart.servier.com/) 14 May 2019.

Local inflammation leads to lymph node swelling (also termed lymph node hypertrophy, reactive lymph node or lymph node shut down), which is a well-known reaction to immune activation, and has been described to be dependent on S1P1, TNF, IFN α/β and CD69 [13,14,15,16]. This phenomenon has been mainly studied in viral and bacterial infections and in terms of the fundamental biology of the chemokine dependency of immune reactions. It involves the active recruitment and delayed exit of immune cells, effectively increasing the lymph node’s cellularity and increasing the chance of cognate interactions between T cells and antigen presenting cells such as dendritic cells (DC). However, also in cancer, TDLN swelling has been reported [17], but its functional relevance has never been investigated. We found an increased swelling of TDLN in contrast to NDLN shortly after PD-1 and PD-L1 blocking treatment in our pre-clinical mouse-models, a clear indication of ongoing immune responses [2]. We hypothesize that this detectable swelling is instrumental in bringing together tumor-antigen presenting DC with specific T cells that may further proliferate in these lymphoid organs. It is imaginable that cognate interaction between DC presenting tumor antigen and specific T cells within TDLN, further enhanced by PD-1/PD-L1 blocking antibodies, contributes to anti-tumor immunity, leading to a greater pool of tumor-specific T cells available for tumor eradication (Figure 1).

Surgical resection may abruptly deplete an important source of tumor-eradicating immune cells counteracting the intended positive effects of the immunotherapy. Recent findings have indicated that immunotherapy efficacy is based on the influx of newly primed T cells into the tumor microenvironment, suggesting a non-stop process, otherwise known as the cancer immunity cycle (a term coined by Chen and Mellman [1]), in these patients [18,19]. This points heavily at the involvement of tumor-draining lymph nodes, where tumor-antigens are being presented, and newly recruited T cells may be primed and additionally activated by the checkpoint blockade, the acceleration of which may be associated with clinical response [20].

## 3. Trafficking to and from TDLN

Although the influence of TDLN on the anti-tumor immune response and, in particular, the efficacy of immunotherapy of cancer have been generally underestimated, there is a great body of research on the lymph drainage of tumors, its role in metastases dissemination and edema problems after resection [6,21].

Besides metastasizing tumor-cells, many other materials such as tumor debris and secreted extracellular vesicles are drained from the tumor to the lymph node. In addition, soluble factors such as chemokines and cytokines have been reported to play a role in creating an immunosuppressive environment as well as changing the stromal cells of the lymph node [18,19,21,22]. Beyond these factors, it was described that a growing tumor physically impacts lymphatics and sentinel lymph nodes such that the architecture and chemokine (receptors) of TDLNs are different from NDLN [19]. Moreover, the permeability of lymphatics is increased, interstitial flow and pressure is enhanced and lymph node size increases, a phenomenon called ‘sinusoidal hyperplasia’ by pathologists [23]. How all these factors influence the immune response against tumors, particularly in light of immunotherapy, remains to be studied in great detail.

Two recent studies showed the importance of lymphatic drainage for the immunotherapy of cancer [24,25]. The overexpression of the soluble factor *VEGF-C*, which is known to increase lymph angiogenesis and increase the risk of metastasis, also strongly increased the anti-tumor effect of immunotherapy with PD-1 checkpoint blockade therapy. Furthermore, it was shown that presence of lymphatic endothelial cells (LEC) within tumors made them more responsive to PD-1 therapy, indicating a clear link between lymph system and checkpoint blockade therapy.

Studies with the so-called “photoconversion” mice, in which the immune cells in tumors under the skin are fluorescently labelled in vivo by UV irradiation, demonstrated immune cell migration from the tumor to TDLN [26,27]. These studies revealed the highly dynamic trafficking of T cells to TDLN, with much more vigorous velocity than previously assumed. A large percentage of T cells that were present in the tumor at the moment of UV irradiations were found in TDLN 24h later. As analyses of TME are always a snapshot, these dynamics should be taken into consideration when drawing conclusions about the abundance of TILs.

## 4. The Effect of Other Treatment on TDLN

The success of checkpoint therapy is undisputed, but still, only a minority of patients have lasting benefits from the treatment. Therefore, combining the checkpoint blockade with other therapy modalities in an attempt to increase clinical benefit is tantamount. The most likely candidates for combinatorial treatments are the already used standard treatments, such as radiotherapy and chemotherapy. In order to implement these combinatorial strategies into daily clinical practice, it is vital to understand the effect these therapy modalities have on the immune function in space and time in order to design rational strategies. Currently, knowledge on the effects radiotherapy and chemotherapy have on the immunological function of the TDLN is limited (Table 1).

Radiotherapy can have a profound effect on tumor cells but can also influence healthy surrounding tissues, including TDLN. Specific doses of radiotherapy will lead to the immunogenic cell death of tumor cells, i.e., the combined release of antigens and damage-associated molecular patterns (DAMPs), which can subsequently lead to DC activation, enhanced tumor antigen uptake and presentation and increased systemic anti-tumor T cell responses. The development of potent anti-tumor T cell responses is dependent on TDLN being able to perform their immunological function, as this is where the tumor antigen would drain to and where uptake and presentation will occur. Since TDLN are under the direct influence of radiotherapy due to their location, the influence of radiotherapy could be crucial to the induction of anti-tumor immune responses, and knowledge about this effect is important for future clinical study designs.

Most studies into the immunological effects of radiotherapy focus on the TME or on subsequent peripheral blood responses [36,37]. Battaglia et al. published a study on the immune content of TDLN after the radiotherapy of cervical cancer patients [35]. And a recent mouse study did point to the importance of TDLN in priming young stem-like effector T cells upon radiotherapy, which, combined with the PD-1 blockade, ensured a subsequent systemic response against non-irradiated tumors (i.e., the abscopal effect) [38].

Both of these studies point at the importance of understanding the effect radiotherapy has on TDLN in order to make rational combinations with immunotherapy.

Similarly, there is little knowledge on the effects of chemotherapy on the immunological function of TDLN. Most chemotherapy agents cause lymphodepletion, as extensively shown in preclinical models and patients. However, this information is based largely on the analysis of peripheral blood. It is likely that similar processes are occurring inside lymph nodes—particularly in TDLN. However, this remains to be studied. Additionally, high doses of glucocorticoids, a common agent given to chemotherapy-treated patients to attenuate nausea and vomiting, severely hampers the antigen presentation by DC and suppresses the secretion of inflammatory cytokines such as IFNα/β and IL-1α/β, as in vitro and in vivo studies have shown [39]. Special interest should go to studying the effect of standard treatment (chemotherapy and/or radiotherapy) on cells that are mostly permanent occupants of lymph nodes, such as endothelial and non-endothelial stromal cells, resident DC, follicular helper cells and fibroblasts, as they may react differently from the peripheral counterparts and are instrumental in the organization of the immune function of lymph nodes.

## 5. Tumor Microenvironment

At present, the majority of studies into the mechanism of checkpoint blocker success are aimed at the TME. There is a wealth of knowledge on the cells and molecules involved in the mechanisms behind the success of checkpoint blocker therapy [40,41]. At present, the general consensus is that PD-1 and PD-L1 blocking antibodies are effective by (re-) activating exhausted T cells, particularly stem-cell-like, “progenitor” exhausted T cells, expressing PD-1, slamF6, Tcf1 but not TIM-3 (to distinguish from “terminally differentiated” exhausted T cells) [42,43,44] and CXCR5 [45]. The paper of Siddiqui [43] describes that for the PD-1 treatment of preclinical mouse-models of cancer, treatment efficacy is solely dependent on TILs and independent of TDLN presence. They employ T cell receptor (TCR) transgenic T cells for their model-experiments, thereby strongly increasing the precursor frequency and number of TILs, possibly explaining why the TDLN does not add to the therapeutic efficacy. The activation of these T cells by checkpoint blockers is dependent on BatF3+ DC and CD28-B7 interactions [46] and involves the secretion of IFN-γ by T cells and IL-12 by DC [47,48,49] within the TME. Recent publications have shown the important role of DC in the PD-1/PD-L1 based anti-tumor T cell inhibition, which can be reversed through PD-1 and PD-L1 blocking antibody therapy [3,50,51]. These interactions could very well take place in TDLN as well, as all components are present in TDLN. This will, however, require detailed evaluation. Analyses of material available from the resections of patients treated with neo-adjuvant therapy will give a great deal of insight into the activation taking place in TME after anti-PD-1 injection [52,53]. So far, in glioblastoma patients treated with neo-adjuvant anti-PD-1, the TME was analyzed after treatment and compared to biopsies from untreated patients or from matched pre-treatment biopsies. Tumor-samples showed enhanced chemokine expression, increased immune infiltrate and augmented TCR clonality compared to pre-treated or non-treated tumor samples [52]. Unfortunately, despite the availability of TDLN material after PD-1 injection, analyses of this organ are not mentioned. A recent study by Thommen et al. described PD-1^high^ cells within tertiary lymphoid structures (TLS) to be implicated with good prognosis after PD-1 therapy in lung cancer patients [54]. The presence of TLS is not always analyzed in TME studies, so it is possible that PD-1^high^ cells commonly reside within lymphoid structures—even lymphoid structures organized within the tumor tissue.

## 6. Neo-Adjuvant Studies

In recent years, the number of publications on neo-adjuvant immunotherapy trials has increased exponentially. It is clear that administering checkpoint blockade in earlier stages of disease, before the resection of tumor and/or TDLN, is considered by many as a potentially efficacious treatment option that warrants extensive evaluation. In the many papers describing, the outcomes and translational research performed on patients undergoing neo-adjuvant immunotherapy, the immunological changes in TME and peripheral blood were meticulously analyzed and described. However, the immune contexture of TDLN was not studied or discussed [53,55,56,57,58,59,60,61,62]. Together, these studies show that neo-adjuvant immunotherapy is feasible and safe, proven by the fact that the planned date of surgery was achieved for the majority of patients. Furthermore, a large proportion of these patients showed complete pathological response (pCR). In glioblastoma patients, neo-adjuvant pembrolizumab (PD-1) administration followed by surgery and continued adjuvant treatment was compared to surgery and adjuvant treatment alone [63]. A significantly extended overall survival combined with higher T cell numbers and the upregulation of an IFN-γ gene expression profile were observed in the patient group receiving neo-adjuvant treatment. In a trial with NSCLC patients, however, a far greater level of adverse events was found than seen in end-stage patients treated with the same regimen, possibly due to the patient’s lower disease burden and greater overall fitness at the time of the start of immunotherapy [56]. This poses a threat to the success rate of the neo-adjuvant use of immunotherapies. A solution might lie in local treatment, which has been explored in recent years and has been found to be very effective, reducing side-effects compared to systemic administration [64,65,66,67,68]. These strategies are very attractive, especially for patients in an earlier stage of the disease, where there is only one identified lesion and no apparent metastases [69]. Experimentally, we have shown significant abscopal immune effects of local treatment to secondary, distant tumors [66,70]. Localized therapy raises the question of which factors need to be present, locally, for treatment to be effective. So far, the general concept has been that checkpoint blocking antibodies target the TME, but we postulate that the presence of TDLN at the time of treatment is an underestimated important factor for therapeutic success, and we hypothesize that this could be an explanation for the increased therapeutic effects seen in neo-adjuvant over adjuvant treated patients. A recent paper by Francis et al. shows that the efficient drainage of immunotherapy to TDLN leads to a superior anti-tumor immune response compared to the administration of checkpoint therapy intratumorally or injection in an area draining to ipsilateral lymph nodes [3]. Furthermore, a study by Koster et al. shows that in patients with stage I-II melanoma, the injection of CpG (a Toll-like Receptor Ligand) into the scar of the recently resected primary tumor leads not only to enhanced immune-activation of the antigen-presenting cells in the TDLN but, more importantly, to enhanced peripheral anti-tumor T cell responses and strongly reduced recurrence and metastases [33]. These two manuscripts both indicate that effective immune-responses that can be (re)activated with immunotherapy can be present within TDLN.

Not only is the neo-adjuvant administration of immunotherapy an excellent way to exploit the full potential of the immune cells within TME and TDLN but it also forms a great source of material for the scientific advancement of the knowledge of TDLN in terms of tumor immunology. Few of the neo-adjuvant studies published at present mention the TDLN. We want to encourage all scientists and clinicians involved in neo-adjuvant trials of immunotherapy to include the TDLN in their immune-monitoring schemes and amplify the current knowledge on the potential of the TDLN for antitumor immunity. Important issues, outstanding at present, are highlighted in Table 1, including the little literature that is available on the subject.

## 7. Conclusions

Tumor draining lymph nodes play a role in cancer that goes far beyond the dissemination of metastases. The contribution of TDLN to the immunotherapy of cancer, particularly the treatment with checkpoint blockers, is slowly becoming apparent. At present, there are 154 clinical trials reported for the neo-adjuvant therapy of immunotherapy, clearly marking the next big step in the implementation of these antibodies. The role of TDLN as the orchestrator of anti-tumor immune responses is becoming more and more apparent, especially in PD-1/PD_L1 checkpoint blockade therapy.

Based on recent scientific findings, as reviewed in this manuscript, we propose a serious re-evaluation of the role of the tumor-draining lymph node in relation to the modes of action of the different immune checkpoint antibodies that are now in the clinic and the possibility to make efficacious treatment combinations. We advocate the meticulous study of all aspects of TDLN immunology, thereby optimally exploiting the anti-tumor immunity present in these specialized lymphoid organs.

## Figures and Tables

**Table 1:** Outstanding questions on the immunological function of TDLN.

	Knowledge Needed	Available Literature
Tumor draining versus healthy lymph nodes	Does the architectural change brought about by growing the tumor change the immunological function of lymph nodes? How strong is the influence of the growing tumor on lymph nodes with descending order of drainage? When is a lymph node still considered tumor-draining?	van Pul et al. 2020 [28] Cochran et al. 2006 [7] Sanchez-Paulete et al. 2017 [8] Gibert-Ramos et al. 2019 [29] Setiadi et al. 2010 [30]
Effect of metastases on Lymph Node	Does the presence of metastatic tumor in the lymph node change the architecture or immunological function?	Ma et al. 2012 [31] van de Ven et al. 2017 [11] Van den Hout et al. 2017 [32]
Difference between lymph nodes draining primary versus metastatic tumors	Do metastasized tumors have a different effect on TDLN than primary tumors? Are the tissues of localization of primary versus metastatic tumors of influence on the architecture and function of lymph nodes?	Ma et al. 2012 [31]
Effect of immunotherapy of lymph nodes	Does the administration of different types of immunotherapy change TDLN?	Koster et al. 2017 [33] Fransen et al. 2018 [2] Dammeijer et al. 2020 [3] Francis et al. 2020 [4]
Effect of other therapies on lymph nodes	Do other standard therapies (chemotherapy, radiotherapy) influence the structure and function of lymph nodes?	Kaewkangsadan et al. 2017 [34] Battaglia et al. 2010 [35]
